# Supplementary material for: Determinants of acceptability of schistosomiasis mass drug administration among primary school children in Busega District, Northwestern Tanzania
Source: PLoS One. 2025 Jul 18;20(7):e0327737. doi: 10.1371/journal.pone.0327737 (PMC12273910; doi:10.1371/journal.pone.0327737)
Supplement: S1 File — This file contains the complete version of the study questionnaire translated into Swahili, which was used for data collection. (DOCX) [file pone.0327737.s001.docx]

CHUO KIKUU CHA ZAMBIA

SKULI YA AFYA YA JAMII

IDARA YA EPIDEMIOLOGY NA BIOSTATISTICS

**KICHWA CHA UTAFITI:** Sababu zinazohusiana na Kukubalika kwa utowaji wa dawa za kichocho miongoni mwa watoto wa shule za msingi katika wilaya ya Busega, Kaskazini magharibi, Tanzania.

Namba ya Dodoso…………………………………………………..

Tarehe ya mahojiano………………………………………………...

Jina la mtafiti ………………………………………………………..

Jina la kata……………………………………………………………

Jina la shule…………………………………………………………..

Mahala shule ilipo……………………………………………………

**MAELEKEZO**

- Dodoso hili lina sehemu tatu (4), sehemu A, B, C na D
- Tafadhali fanya mahojiano kwa watoto wa shule umri kati ya miaka 10-17
- Zungushia jibu lililosahihi
- Majibu yote yatunzwe kwa usiri
- Hakikisha kuwa maswali yote yanajibiwa

**Sehemu A: Takwimu za kidemografia na jamii**

1. Wewe ni jinsia gani?
2. Mwanamme
3. Mwanamke
4. Una umri gani/mwaka wako wa kuzaliwa……………………
5. Uko darasa la ngapi? .............................................
6. Chanzo cha mapato cha wazazi wako ni kipi? (kichwa cha familia) ……………………
7. Kiwango cha elimu cha wazazi wako ni kipi? (kichwa cha familia)
8. Hawajasoma
9. Elimu ya msingi
10. Elimu ya sekondari
11. Elimu ya ufundi
12. Stashahada/Shahada
13. Sifahamu
14. Unasali dhehebu gani?
    1. Kanisa Catholic
    2. Kanisa la Anglikana
    3. Msabato
    4. Makanisa ya Kilokole
    5. Muislamu
    6. Kanisal la AIC (African-initiated church)
    7. Kanisa la kiinjili la kilutheri Tanzania
    8. Kanisa la Efata
    9. Hauna dini

Nyingine (taja)……………………………

1. Kabila lako ni lipi?...........................................
2. Je unaishi karibu na chanzo cha maji?
3. Ndiyo
4. Hapana
5. Kama jibu ni ndiyo kwenye swali la (8) hapo juu, ni vyanzo vipi?
6. Dimbwi
7. Mto
8. Bwawa
9. Ziwa

Kingine (taja) ------------------------

1. Umeshawahi kumeza hizi dawa (mshiriki kuonyeshwa praziquantel tablets)
2. Ndio
3. Hapana

**Sehemu B: Hali ya Ulemavu wa mshiriki (Vipengele 6)**

1. Je, unapata ugumu wowote wa kuona, hata kwa kuvaa miwani?
2. Hapana sipati ugumu
3. Ndiyo, Napata ugumu kiasi
4. Ndiyo, Napata ugumu sana
5. Siwezi kabisa kuona
6. Sipendelei kusema
7. Nisingependa kusema Je, unapata ugumu kusikia, hata kwa kutumia kifaa cha kukusaidia kusikia?
8. Hapana sipati ugumu
9. Ndiyo, Napata ugumu kiasi
10. Ndiyo, Napata ugumu sana
11. Siwezi kabisa kusikia
12. Sipendelei kusema
13. Je, Unapata ugumu kupanda ngazi au kupiga hatua?
14. Hapana sipati ugumu
15. Ndiyo, Napata ugumu kiasi
16. Ndiyo, Napata ugumu sana
17. Siwezi kabisa kutembea au kupanda ngazi
18. Sipendelei kusema
19. Je, Unapata ugumu wowote kukumbuka au kuzingatia?
20. Hapana sipati ugumu
21. Ndiyo, Napata ugumu kiasi
22. Ndiyo, Napata ugumu sana
23. Siwezi kabisa kukumbuka au kuzingatia
24. Sipendelei kusema

5. Je, Unapata ugumu wowote (kujihudumia au kujijali kama vile) kuoga au kuvaa?

1. Hapana sipati ugumu
2. Ndiyo, Napata ugumu kiasi
3. Ndiyo, Napata ugumu sana
4. Siwezi kabisa
5. Sipendelei kusema

6. Kwa kutumia lugha uliyoizoea, Je Unapata ugumu wowote kuwasiliana (kwa mfano kuelewa au kueleweka na wengine)?

1. Hapana sipati ugumu
2. Ndiyo, Napata ugumu kiasi
3. Ndiyo, Napata ugumu sana
4. Siwezi kabisa
5. Nisingependa kusema

**Sehemu C: Hali ya kukubalika kwa utowaji wa dawa za kichocho kwa wingi**

1.Je dawa za kichocho zina mvuto wowote kwako?

1. Sikubali kabisa
2. Sikubali
3. Sina maoni
4. Nakubali
5. Nakubali sana

2.Unapingamizi yeyote inayoweza kukufanya usimeze hizi dawa za kichocho?

- 1. Sikubali kabisa
  2. Sikubali
  3. Sina maoni
  4. Nakubali
  5. Nakubali sana

3.Je dawa hizi za kichocho unazipenda?

- 1. Sizipendi hata kidogo
  2. Sizipendi
  3. Sina maoni
  4. Nazipenda
  5. Nazipenda sana

4.Je unahisi ni sawa kwako kuchukua/kumeza dawa za kichocho?

1. Ndio
2. Hapana

5.Kulingana na mtazamo wako, Je unahisi dawa za kichocho (Praziquantel) ni nija nzuri ya kuzuia ugonjwa wa kichocho?

- 1. Sikubali kabisa
  2. Sikubali
  3. Sina maoni
  4. Nakubali
  5. Nakubali sana

6.Je unaridhika na umezaji/ uchukuji wa dawa za kichocho kama njia ya kujikinga na kichocho?

1. Siridhishwi nao kabisa
2. Siridhishwi nao
3. Sina maoni
4. Naridhishwa nao
5. Narifhishwa nao sana

**Sehemu D: Sababu zinazopelekea kukubalika kwa ugawaji wa dawa za kichocho kwa wingi miongoni mwa watoto wa shule za msingi**

1.Je unajisikia vipi mtu akikuambia kumeza dawa za kichocho (praziquantel)?

1. Vibaya sana
2. Vibaya
3. Sina maoni
4. Vizuri
5. Vizuri sana

2. Je, unafikiri dawa za kichocho zinaweza kuboresha/kuongeza uwezekano wowote wa wewe kutokuwa mgonjwa wa kichocho?

1. Sikubali kabisa

2. Sikubali

3. Sina maoni

4. Nakubali

5. Nakubali sana

3.Je unaelewa ni kwa jinsi gani ukimeza dawa za kichocho (praziquantel) zinapunguza hatari ya wewe kuugua kichocho?

- 1. Sielewi kabisa
  2. Sielewi
  3. Sina maoni
  4. Naelewa
  5. Naelewa sana

4.Je Unadhani kuna makosa yeyote unayoyafanya endapo utachukua au kumeza dawa za kichocho?

1. Sikubali kabisa
2. Sikubali
3. Sina maoni
4. Nakubali
5. Nakubali sana

5. Je unadhani kuwa ni haki kwako kumeza dawa za kichocho (praziquantel)?

1. Sio haki kabisa
2. Siyo haki
3. Sina maoni
4. Ni haki
5. Ni haki sana

6.Je unatakiwa kusafiri kwa umbali mrefu ili uweze kupata dawa za kichocho?

1. Ndio
2. Hapana

7. Je unatakiwa kusubiri kwa muda mrefu ili uweza kupata dawa za kichocho?

1. Ndio
2. Hapana

8.Je inachukua juhudi kiasi gani kwako ili kupokea dawa za kichocho?

1. Hakuna juhudi hata kidogo
2. Juhudi kidogo
3. Sina maoni
4. Juhudi nyingi
5. Juhudi kubwa

9.Je unaamini kuwa dawa za kichocho ni salama na malengo yake ni kukukinga ili usipate ugonjwa wa kichocho?

1. Sikubali kabisa
2. Sikubali
3. Sina maoni
4. Nakubali
5. Nkubali sana

10. Je unajiamini kiasi gani kuwa unaweza kufanya kile kinachohitajika kuchukua/kumeza dawa za kichocho?

1. Sijiamini kabisa

2. Sijiamini

3. Sina maoni

4. Najiamini

5. Najiamini sana

11.Je, kujihusisha na uchukuaji wa dawa za kichocho unaingilia kazi zako za shule au kazi zingine?

1. Sikubali kabisa
2. Sikubali
3. Sina maoni
4. Nakubali
5. Nakubali sana
