# Supplementary material for: Determinants of acceptability of schistosomiasis mass drug administration among primary school children in Busega District, Northwestern Tanzania
Source: PLoS One. 2025 Jul 18;20(7):e0327737. doi: 10.1371/journal.pone.0327737 (PMC12273910; doi:10.1371/journal.pone.0327737)
Supplement: S2 File — This file provides the original English version of the questionnaire used in the study. (DOCX) [file pone.0327737.s002.docx]

THE UNIVERSITY ZAMBIA

SCHOOL OF PUBLIC HEALTH

DEPARTMENT OF EPIDEMIOLOGY AND BIOSTATISTICS

**RESEARCH TITTLE**: Determinants of acceptability of schistosomiasis mass drug administration among school children in Busega District, Northwestern Tanzania

IDENTIFICATION INFORMATIONS

Questionnaire ID/Code……………………… ………………………….

Date of interview…………………………………………………………

Name of interviewer…………………………. ………………………….

Name of Ward…………………….…………... ………………………….

Name of school…………………………………………………………….

Geographical location of the school………………………………………..

**INSTRUCTIONS**

- This questionnaire consists of Four sections, section A, B, C and D
- Please only interview pupils between age 10-17
- Please circle where applicable
- All the responses remain confidential
- Interviewer to countercheck if all questions are well answered

**Section A: Background characteristics of the study participants**

1. What gender are you?
2. Male
3. Female
4. How old are you? …….……………………
5. Which class are you? .............................................
6. What is your parent/guardian source of income (head of family) …………….?
7. What is your parent’s/guardian level of education? (Head of family)
8. No education
9. Primary school
10. Secondary school
11. Technical education
12. Diploma/Degree
13. I don’t know
14. What religion do you practice?
    1. Catholic
    2. Anglican
    3. Seventh Day Adventist
    4. Evangelism Assemblies of God Tanzania (EAGT)
    5. Muslim
    6. African-initiated church (AIC)
    7. Kanisa la kiinjili la kilutheri Tanzania (KKKT)
    8. EFATA ministries
    9. No religion

Others, specify……………………….

1. Which tribe are you? ……....................
2. Are you living nearby any water body?
3. Yes
4. No
5. If ‘yes’ to questions (8) above, what is the name of the water body
6. Pond
7. River
8. Dam
9. Lake

Other (specify) ------------------------

1. Have you ever swallowed this tablet (Respondent will be shown a sample of praziquantel tablets)?
2. Yes
3. No

**Section B: Individual Disability status (Items: 6)**

Washington Group Short Set of Disability Questions

*Everyone’s body and mind work differently. In the next few questions, we’d like to learn about how your body/mind works. We would like to start by asking about difficulties you may have doing certain activities because of a health problem. Please remember that this information is confidential and you may choose to skip any question you do not want to answer.*

1. Do you have difficulty seeing, even if wearing glasses?
   1. No, no difficulty
   2. Yes, some difficulty
   3. Yes, a lot of difficulty
   4. Cannot do it at all
   5. Prefer not to say
2. Do you have difficulty hearing, even if using a hearing aid?
   1. No, no difficulty
   2. Yes, some difficulty
   3. Yes, a lot of difficulty
   4. Cannot do it at all
   5. Prefer not to say
3. Do you have difficulty walking or climbing steps?
   1. No, no difficulty
   2. Yes, some difficulty
   3. Yes, a lot of difficulty
   4. Cannot do it at all
   5. Prefer not to say
4. Do you have difficulty remembering or concentrating?
   1. No, no difficulty
   2. Yes, some difficulty
   3. Yes, a lot of difficulty
   4. Cannot do it at all
   5. Prefer not to say
5. Do you have difficulty (with caring for yourself such as) bathing or dressing yourself?
   1. No, no difficulty
   2. Yes, some difficulty
   3. Yes, a lot of difficulty
   4. Cannot do it at all
   5. Prefer not to say
6. Using your usual language, do you have difficulty communicating (for example understanding or being understood by others)?
   1. No, no difficulty
   2. Yes, some difficulty
   3. Yes, a lot of difficulty
   4. Cannot do it at all
   5. Prefer not to say

**Section C: Acceptability of schistosomiasis mass drug administration (Items: 6)**

1. Is Schistomiasis drugs pleasing to you?

1. Strongly disagree
2. Disagree
3. No opinion
4. Agree
5. Strongly agree

2. Do you have any objections to take schistosomiasis drugs?

1. Strongly agree
2. Agree
3. No opinion
4. Disagree
5. Strongly disagree

3.Do you like the schistosomiasis drugs (Praziquantel)?

1. Don’t like at all
2. Don’t like it
3. No opinion
4. Like it
5. Like it a lot

4.Are you okay with taking schistosomiasis drugs (praziquantel)

1. Strongly disagree
2. Disagree
3. No opinion
4. Agree
5. Strongly agree

5.According to you, is schistosomiasis drugs (Praziquantel) a good medication**/**intervention?

1. Strongly disagree
2. Disagree
3. No opinion
4. Agree
5. Strongly agree

6. Is this schistosomiasis drug a satisfactory medication**/**intervention to you?

1. Very unsatisfactory
2. Unsatisfactory
3. No opinion
4. Satisfactory
5. Very satisfactory

**Section D: Determinants associated with acceptability of schistosomiasis mass drug administration.**

1. How comfortable do feel you can swallow schistosomiasis drugs (praziquantel tablets)?

1. Very uncomfortable
2. Uncomfortable
3. No opinion
4. Comfortable
5. Very comfortable

2. Do you think schistosomiasis drugs is likely to improve any chance of you not becoming ill from schistosomiasis?

1. Strongly disagree
2. Disagree
3. No opinion
4. Agree
5. Strongly agree

3. Is it clear to you how swallowing praziquantel will reduce the risk of you becoming ill from schistosomiasis?

1. Strongly disagree
2. Disagree
3. No opinion
4. Agree
5. Strongly agree

4. Do you think your committing wrong for you to take schistosomiasis drugs (praziquantel tablets)?

1. Strongly disagree
2. Disagree
3. No opinion
4. Agree
5. Strongly agree

5.Taking/ swallowing schistosomiasis drugs (praziquantel tablets) is it fair to you?

1. Very unfair
2. Unfair
3. No opinion
4. Fair
5. Very fair

6. Did you have to travel for a long distance in order for you to receive schistosomiasis drugs?

1. Yes
2. No

7. Did you have to wait for a long time in order for you to receive schistosomiasis drugs?

1. Yes
2. No

8.How much effort did it take to receive schistosomiasis drugs?

1. No effort at all
2. A little effort
3. No opinion
4. A lot of effort
5. Huge efforts

9.Do you think that schistosomiasis drugs (praziquantel tablets) are safe, and their aim is to prevent from becoming ill from schistosomiasis?

1. Strongly disagree
2. Disagree
3. No opinion
4. Agree
5. Strongly agree

10. How confidence did you that you can do what is required to take praziquantel?

1. Very unconfident
2. Unconfident
3. No opinion
4. Confident
5. Very confident

11. Engaging in receiving schistosomiasis drugs (praziquantel) interfered with your school activities or any other activities?

1. Strongly disagree
2. Disagree
3. No opinion
4. Agree
5. Strongly agree
