# Supplementary material for: Determinants of acceptability of schistosomiasis mass drug administration among primary school children in Busega District, Northwestern Tanzania
Source: PLoS One. 2025 Jul 18;20(7):e0327737. doi: 10.1371/journal.pone.0327737 (PMC12273910; doi:10.1371/journal.pone.0327737)
Supplement: Attachment — Submitted filename: Response to Reviewers.docx [file pone.0327737.s006.docx]

The Editor

PLOS ONE

June 10, 2025

RE: Resubmission of revised manuscript entitled “Determinants of acceptability of schistosomiasis mass drug administration among primary school children in Busega District, Northwestern Tanzania”

Thank you for your positive and helpful comments on our manuscript. Kindly find below the response to the comments that were raised by the reviewers.

Point-by point responses (in blue) to the comments raised by reviewers are shown below. A 'clean' and 'tracked' version of our revisions have been uploaded; the tracked version indicating the changes made to the manuscript during revision.

Sincerely,

Irene Yunzu Edward

[ireneyunzu@gmail.com](mailto:ireneyunzu@gmail.com)

**ACADEMIC EDITOR**

1. The two reviewers have made some suggestions for improvement of the manuscript. Overall, these are minor edits and should help to improve the manuscript.

Thanks for taking time to read the manuscript and sharing your valuable comments.

2. The manuscript has merit and is well written. I suggest a minor edit under the Methods on theoretical framework with respect to the definition of intervention coherence- check the grammar and revise.

Thank you for the comment. We have revised the definition for clarity. It now reads: **Intervention coherence is defined as the extent to which participants understand the purpose of the intervention (MDA) and how it works (*Page 5, lines 130–132)*.**

**Additional Editor Comments**

The study is titled 'Determinants of acceptability of schistosomiasis mass drug administration among primary school children in Busega District, Northwestern Tanzania' and sought to establish factors associated with acceptability of schistosomiasis mass drug administration among primary school children in Busega District. The objectives, methods, results and discussions are sufficiently done but kindly take care to revise the the manuscript based on the comments of the reviewers.

Thanks for taking time to read the manuscript and sharing your valuable comments

One other very minor correction: Under the theoretical framework, the sentence 'Intervention coherence related to the extent to which the participant understands the purposes of MDA' seems incomplete. Kindly revise. Consider 'Intervention coherence is....................................

Thank you for the comment. We have revised the definition for clarity. It now reads: **Intervention coherence is defined as the extent to which participants understand the purpose of the intervention (MDA) and how it works (*Page 5, lines 130–132)*.**

**REVIEWERS' COMMENTS:**

**REVIEWER REPORTS**

**Reviewer #1:**

1.This is an interesting social study on Praziquantel MDA.
I recommend authors to add more details on how the children consented, whether it was individually or not.

Thank you for the comment. We have added this information in the ethical considerations section (*Pages 9, lines 258–260).*

Assent forms were provided to eligible primary school children. The consent process was conducted individually, whereby each child who agreed to participate signed an assent form.

2. Additionally, since MDA is done to all SAC i.e. 5 years and above (till end of Primary school), there is a need to explain why authors chose to include only those in grade V-VII in the study.

Thank you for the comment. Although MDA targets all school-age children (SAC) aged 5 years and above, this study focused on students in grades V–VII for several considerations. First, these students had the full benefit of two to three rounds of MDA (2018, 2019, and 2021), providing sufficient exposure to evaluate their experiences. In addition, older students are more likely to comprehend and articulate their experiences, perceptions, and concerns regarding the intervention, which is critical in assessing acceptability. This approach also aligns with similar studies on MDA, which have prioritized upper primary students (26,27).

We have explained this in detail in study population section *(Page 6, lines 166-175).*

3. Add when MDA (year) in reference was conducted? I just see that data collection time March- May 2023.The gap between the two timelines could contribute to some variables in question.

Thank you for the comment. The last round, which MDA was conducted in this district was 2021. No mass praziquantel treatment was administered in 2022, a year before data collection for this study was done *(Page 5, lines 147-148).*

**Reviewer #2:**

1. Title: Determinants of acceptability of schistosomiasis mass drug administration among primary school children in Busega District, Northwestern Tanzania.
Introduction: Kindly rephrase this statement for clarity “Acceptability is a multi-faceted construct that reflects the extent to which receiving an intervention consider it to be appropriate, based on anticipated or experienced cognitive and emotional responses to the intervention.

Thank you for the comment. We have revised the definition to make it clearer. It now reads: Acceptability is the perception among implementation stakeholders that a given treatment, service, practice or innovation is agreeable, palatable, or satisfactory *(Page 4, lines 105-107).*
